# Severe Acute Kidney Injury Following Whipple Procedure

**DOI:** 10.34067/KID.0000001078

**Published:** 2026-05-28

**Authors:** Hamza Akel, Shivangi K. Patel

**Affiliations:** 1 Rowan-Virtua School of Osteopathic Medicine, Stratford, New Jersey; 2 Department of Nephrology, Morristown Medical Center, Morristown, New Jersey

**Keywords:** AKI, chronic hemodialysis, kidney biopsy

## Abstract

**Figure:**
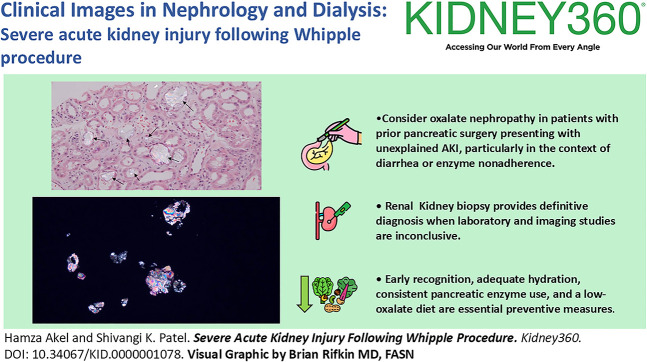

## Case Description

A 76-year-old man with diabetes mellitus, hypertension, and intraductal papillary mucinous neoplasm status following Whipple procedure 6 months earlier presented with severe fatigue, weakness, and shortness of breath for 3 days, accompanied by nausea, anorexia, and 1 day of anuria. He reported several days of watery diarrhea. His medications included lisinopril, metformin, pancrelipase, and occasional nonsteroidal anti-inflammatory drugs.

On admission, BP was 85/51 mm Hg, pulse 70 bpm, with normal oxygen saturation, and decreased skin turgor. Laboratory evaluation revealed creatinine 9.7 mg/dl (baseline 1.3 mg/dl approximately 6 months prior), eGFR 13 ml/min per 1.73 m^2^, bicarbonate 7 mmol/L, potassium 6.7 mmol/L, calcium 7.8 mg/dl, phosphorus 12.9 mg/dl, and venous blood gas 7.09/19/5.8. Urinalysis showed large blood, >50 red blood cells, and 200 dipstick protein. Repeat testing demonstrated trace protein, small blood. Serologies for vasculitis and monoclonal disease were negative. Gastrointestinal PCR was positive for enteroaggregative *Escherichia coli*, and abdominal computed tomography revealed no colitis, stones, or hydronephrosis.

Acute tubular necrosis secondary to volume depletion exacerbated by angiotensin-converting enzyme inhibitor use was initially suspected. Despite correction of metabolic abnormalities, hemodynamics, and resolved diarrhea, he continued to have worsening kidney function and was initiated on dialysis.

**Figure 1:**
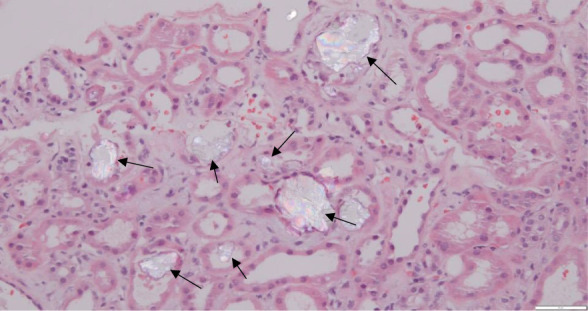
**Kidney biopsy stained with H&E showing oxalate crystal deposits within dilated tubules and increased lymphocytes within the interstitial space, signifying tubular injury.** H&E, Hematoxylin and Eosin.

**Figure 2:**
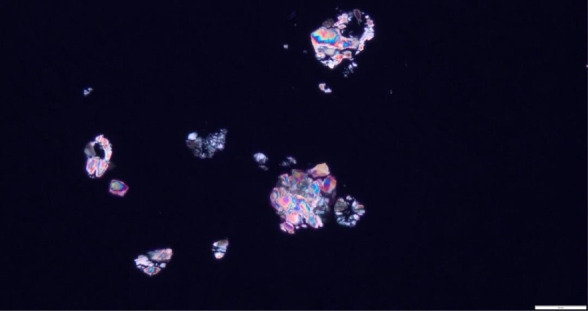
Positive birefringent crystals under polarized light microscopy in clusters confirming oxalate nephropathy.

Kidney biopsy revealed diffuse tubular injury with numerous oxalate crystals, with mild to moderate interstitial fibrosis and tubular atrophy diagnostic of oxalate nephropathy (Figures 1 and 2). A spun urine sediment examination was not performed but could have alluded to the diagnosis. Pancrelipase was increased, and vitamin D_3_ and calcium acetate were initiated with a low-oxalate diet. Despite these interventions, kidney recovery did not occur, and he remained dialysis dependent.

## Discussion

Oxalate nephropathy occurs when high levels of oxalate crystals are deposited in kidney parenchyma, leading to kidney failure.^1^ Hyperoxaluria can be inherited or acquired.

Acquired causes of hyperoxaluria can be categorized into excessive dietary oxalate intake, impaired intestinal oxalate degradation, increased colonic permeability, and most commonly, malabsorptive states such as chronic pancreatitis and gastric bypass.^2,3^ In fat malabsorption, unabsorbed fatty acids bind intestinal calcium, reducing calcium available to bind dietary oxalate. This increases free oxalate absorption resulting in hyperoxaluria and kidney injury.^4^

In this patient, the combination of poor compliance with pancreatic enzyme usage and diarrheal illness amplified intestinal oxalate uptake. Once deposited, oxalate crystals can provoke sustained tubular injury triggering inflammatory response even after the inciting factor resolves, resulting in inflammatory-associated kidney injury explaining the poor kidney recovery observed.^3,5^

Postpancreatic surgery oxalate nephropathy is uncommon but carries a poor prognosis—approximately 1% of such patients develop biopsy-proven disease,^1,2^ and more than half remain dialysis dependent despite corrective therapy.^3^

## Teaching Points

- Consider oxalate nephropathy in patients with prior pancreatic surgery presenting with unexplained AKI, particularly in the context of diarrhea or enzyme nonadherence.
- Kidney biopsy provides a definite diagnosis when laboratory and imaging studies are inconclusive.
- Early recognition, adequate hydration, consistent pancreatic enzyme use, and a low-oxalate diet are essential preventive measures.

## Supplementary Material
