# Supplementary material for: Severe Acute Kidney Injury Following Whipple Procedure
Source: Kidney360. 2026 May 28;7(5):1187–8. doi: 10.34067/KID.0000001078 (PMC13229433; doi:10.34067/KID.0000001078)
Supplement: Supplementary file 1 [file kidney360-7-1187-s001.pdf]

ASN American Society of Nephrology JASN Journal of the American Society of Nephrology KIDNEY360 Accessing Our World From Every Angle CJASN Clinical Journal of the American Society of Nephrology

ASN Journal Disclosure Form

As per ASN journal policy, I have disclosed any financial relationships or commitments I have held in the past 36 months as included below. I have listed my Current Employer below to indicate there is a relationship requiring disclosure. If no relationship exists, my Current Employer is not listed.

H. Akel has nothing to disclose.

I understand that the information above will be published within the journal article, if accepted, and that failure to comply and/or to accurately and completely report the potential financial conflicts of interest could lead to the following: 1) Prior to publication, article rejection, or 2) Post-publication, sanctions ranging from, but not limited to, issuing a correction, reporting the inaccurate information to the authors' institution, banning authors from submitting work to ASN journals for varying lengths of time, and/or retraction of the published work.

Name: Hamza Akel
Manuscript ID: K360-2025-001318R1
Manuscript Title: Severe acute kidney injury following Whipple procedure
Date of Completion: November 13, 2025
Disclosure Updated Date: November 4, 2025

ASN American Society of Nephrology
JASN Journal of the American Society of Nephrology
KIDNEY360 Accessing Our World From Every Angle
CJASN Clinical Journal of the American Society of Nephrology

ASN Journal Disclosure Form

As per ASN journal policy, I have disclosed any financial relationships or commitments I have held in the past 36 months as included below. I have listed my Current Employer below to indicate there is a relationship requiring disclosure. If no relationship exists, my Current Employer is not listed.

S. Patel reports the following:
Employer: Atlantic Health System

I understand that the information above will be published within the journal article, if accepted, and that failure to comply and/or to accurately and completely report the potential financial conflicts of interest could lead to the following: 1) Prior to publication, article rejection, or 2) Post-publication, sanctions ranging from, but not limited to, issuing a correction, reporting the inaccurate information to the authors' institution, banning authors from submitting work to ASN journals for varying lengths of time, and/or retraction of the published work.

Name: Shivangi Patel
Manuscript ID: K360-2025-001318R1
Manuscript Title: Severe acute kidney injury following Whipple procedure
Date of Completion: November 13, 2025
Disclosure Updated Date: November 4, 2025
